# Soret and Dufour Effects on MHD Peristaltic Flow of Jeffrey Fluid in a Rotating System with Porous Medium

**DOI:** 10.1371/journal.pone.0145525

**Published:** 2016-01-25

**Authors:** Tasawar Hayat, Maimona Rafiq, Bashir Ahmad

**Affiliations:** 1 Department of Mathematics, Quaid-I-Azam University 45320, Islamabad, 44000, Pakistan; 2 Nonlinear and Applied Mathematics (NAAM) Research Group, Department of Mathematics, King Abdulaziz University, Jeddah, 21589, Saudi Arabia; Tsinghua University, CHINA

## Abstract

The objective of present paper is to examine the peristaltic flow of magnetohydrodynamic (MHD) Jeffrey fluid saturating porous space in a channel through rotating frame. Unlike the previous attempts, the flow formulation is based upon modified Darcy's law porous medium effect in Jeffrey fluid situation. In addition the impacts due to Soret and Dufour effects in the radiative peristaltic flow are accounted. Rosseland’s approximation has been utilized for the thermal radiative heat flux. Lubrication approach is implemented for the simplification. Resulting problems are solved for the stream function, temperature and concentration. Graphical results are prepared and analyzed for different parameters of interest entering into the problems.

## Introduction

The flow induced by travelling waves along the channel walls has accorded the attention here due to its significance in physiological and industrial applications. Many biological ducts for example the digestive system [1] and the ureter [2], convey their fluid contents by peristalsis. It is also employed for blood transport in capillaries, chyme motion in the gastrointestinal tract, intrauterine fluid motion, movement of ovum in the female fallopian tube etc. In the industry, this mechanism is adopted for controlled transport of fluids inside the tracts. To prevent the blockage and to keep apart the fluid contents from the tract boundaries, the peristalsis also used to provide additional pumping to the flows in heart-lung machines, artificial heart pumps etc. There is ample information on peristalsis now but some recent developments on the topic may be seen through the studies [3–12].

Heat transfer involved many complicated processes in tissues such as heat conduction in tissues, heat convection due to blood flow through pores of the tissues, metabolic heat generation and external interactions such as electromagnetic radiation emitted from electronic devices. The combined effect of heat and mass transfer is mostly useful in the chemical industry and in reservoir engineering in connection with thermal recovery process and may be found in salty springs in the sea. Heat and mass transfer effects are quite prevalent in hemodialysis, oxygen and nutrients diffuse out of the blood vessels to the neighboring tissues. Magnetohydrodynamic in peristalsis is very important physiologically such as the presence of hemoglobin molecule makes the blood a bio-magnetic fluid. Magnetic Resonance Imaging (MRI), magnetic devices and magnetic particles used as drug carriers have some applications of magnetic field in physiology. Further simultaneous occurrence of heat and mass transfer affecting each other lead to the Soret and Dufour effects. Some studies discussing the heat and mass transfer in peristalsis are mentioned in the refs. [13–24].

Most biological and industrial fluids are non-Newtonian in nature. Few examples of such fluids include semisolid food, called bolus, in esophagus, semiliquid food (chyme) in stomach and intestines, reproductive and glandular secretions, flows of metal alloys with oil and grease in automobiles. Due to diverse characteristics of fluids in nature it is not possible to have one constitutive relationship predicting the effects of all non-Newtonian fluids. Therefore many non-Newtonian fluid models have been developed to study the realistic flows like Maxwell's model [25] in blood flows, empirical models [26] for eye fluid dynamics and Oldroyd-B models for embryological transport [27] etc. The Jeffrey liquid amongst these materials is the simplest linear model which discusses the non-Newtonian fluid properties for which one can reasonably hope to obtain exact or analytical solutions.

Fluid flow through porous medium is topic of a great interest due to its applications in the fields of engineering, geo-fluid dynamics and biomechanics. In human physiological systems such flows can be observed in kidneys, lungs, movement of small blood vessels, cartilage and bones etc. Inside human body the tissues can be regarded as deformable porous media. Their working depends on the transport of blood and different nutrients through them. Therefore researchers have modelled the flow of Newtonian/non-Newtonian fluids through porous media in order to observe different diseases like tumor growth. Some recent investigations in this direction can be seen through the studies [28–30].

The phenomenon of rotation has its wide applications in cosmic and geophysical flows. Moreover occurrence of rotation also helps in better understanding the behavior of ocean circulation and galaxies formation. It is also applied to study the nanoparticle orientation in fluid systems through rotational diffusion [31–33]. Rotation also helps in the measurement of the energies of transitions between quantized rotational states of molecules in the gas phase (rotational spectroscopy) [34]. In particular the peristalsis of MHD fluid in presence of rotation is relevant with regard to certain flow cases involving the movement of physiological fluids for example the blood and saline water. Obviously the magnetic field and rotation are useful for biofluid transport in the intestines, ureters and arterioles. Further energy flux induced by concentration gradient is known as thermal diffusion (Dufour) effect. Mass flux can be generated due to temperature gradient (Soret). Although the diffusion-thermo and thermal-diffusion are regarded small order of magnitude in comparison to the influences due to Fourier's or Fick's law but there are situations where such effects cannot be ignored. For example, the thermal diffusion effect is employed for isotope separation and in mixtures between gases with high molecular weight (*H*_*2*_, *He*) and of medium molecular weight (*N*_*2*_, *air*), the diffusion-thermo effect cannot be omitted. Having all such viewpoints in mind the purpose here is to discuss the peristaltic flows of MHD non-Newtonian fluid in a rotating frame. To our knowledge very few attempts are made for peristaltic flows when both fluid and channel are in a state of solid body rotation [35–39]. Here we analyze the effect of rotation on MHD peristalsis of Jeffrey fluid in a porous medium. Soret and Dufour effects are present. Thermal radiation in energy equation is considered. Unlike the traditional approach, the modified Darcy's law is used for the porous medium effect. The relevant equations are modeled and solved through lubrication approach. Results are discussed for related parameters and interpreted physically.

## Modeling

**Fig 1:**
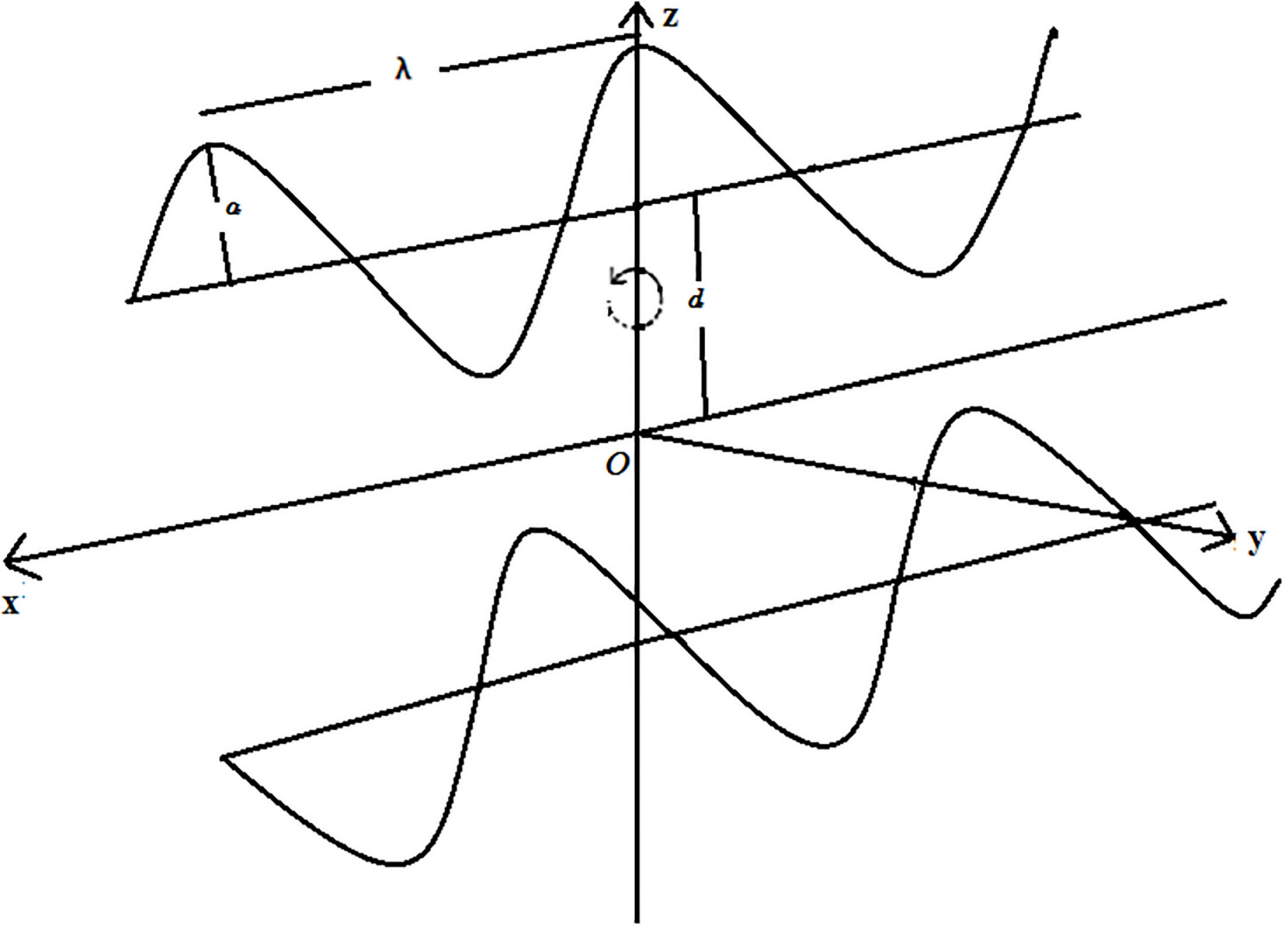
Geometry of the problem.

We examine the peristaltic motion of an incompressible Jeffrey liquid in a compliant wall channel. An incompressible liquid saturates the porous space between the flexible walls of channel. Fluid is electrically conducting due to uniform applied magnetic field of strength *B*_*0*_ Induced magnetic field subject to low magnetic Reynolds number is neglected. Electric field contribution is not taken into account. Effects of Soret and Dufour and thermal radiation are retained. The whole system is in a rotating frame of reference with constant angular velocity ***Ω***. Flow configuration is presented in Fig 1. The channel walls are taken at *z = ±η* Shapes of the travelling waves are described by the following expression:

$$\text{z}=\pm \eta \left(\text{x},\text{t}\right)=\pm [\text{d }+\text{a sin}(\frac{2\pi }{\lambda }\left(\text{x}–\text{ct}\right))]$$ (1)

where *a* depicts the wave amplitude, *t* the time, *d* the half width of channel, *λ* the wavelength and *c* the wave speed. The fundamental equations governing the present flow and heat/mass transfer are represented by

∇.V= 0, (2)

$$\rho \frac{dV}{dt}+\;\rho \left[\Omega \times \left(\Omega \times r\right)\;+\;2\left(\Omega \times V\right)\right]\;=\nabla .\tau +J\times B+R,$$ (3)

$$\rho C_{p}\frac{dT}{dt}\;=\;\kappa \nabla^{2}\text{T }+\tau .L+\frac{DK_{T}}{C_{s}}\nabla^{2}\text{C }-\frac{\partial q_{r}}{\partial z},$$ (4)

$$\frac{dC}{dt}=D\nabla^{2}C+\frac{DK_{T}}{T_{m}}\nabla^{2}T,$$ (5)

$$\begin{array}{l} \tau =-pI+S,\\ S=\frac{\mu }{1+\lambda_{1}}\left(1+\lambda_{2}\frac{d}{dt}\right)A_{1},\\ A_{1}=\;\left(\text{grad}V\right)\;+\;{\left(\text{grad}V\right)}^{\text{transpose}} \end{array}$$ (6)

in which *ρ* shows the fluid density, *p* the pressure, ***I*** the identity tensor, ***S*** the extra stress tensor, ***A***_***1***_ the first Rivlin Erickson tensor, ***τ*** the Cauchy stress tensor, ***Ω*** = *Ωk* the angular velocity, *C*_*p*_ the specific heat at constant volume, κ the thermal conductivity, *T* the temperature of fluid, *D* the coefficient of mass diffusivity, *K*_*T*_ the thermal diffusion ratio, *C*_*s*_ the concentration susceptibility, *C* the concentration of fluid and *T*_*m*_ the mean temperature of fluid, *η* the dynamic viscosity, *λ*_*1*_ the ratio of relaxation to retardation times and *λ*_*2*_ the retardation time.

On the basis of Jeffrey fluid model the expression of Darcy's' resistance is:

$$\text{R}\;=\;-\frac{\mu \Phi }{\text{K}\left(1+\lambda_{1}\right)}\left(1+\lambda_{2}\frac{\text{d}}{\text{dt}}\right)\text{V}$$ (7)

where Φ(0<Φ<1) and K(>0) are respectively the (constant) porosity and permeability of the porous medium. Eqs (2), (3) and (6) yield

$$\frac{\partial u}{\partial x}+\frac{\partial v}{\partial y}+\frac{\partial w}{\partial z}=0,$$ (8)

$$\rho \left[\frac{du}{dt}\right]-2\rho \Omega v=-\frac{\partial p}{\partial x}+\frac{\partial S_{xx}}{\partial x}+\frac{\partial S_{xy}}{\partial y}+\frac{\partial S_{xz}}{\partial z}-\sigma B_{0}^{2}u-\frac{\mu \Phi }{\text{K}\left(1+\lambda_{1}\right)}\left(1+\lambda_{2}\frac{\text{d}}{\text{dt}}\right)u,$$ (9)

$$\rho \left[\frac{dv}{dt}\right]+2\rho \Omega v=-\frac{\partial p}{\partial y}+\frac{\partial S_{yx}}{\partial x}+\frac{\partial S_{yy}}{\partial y}+\frac{\partial S_{yz}}{\partial z}-\sigma B_{0}^{2}v-\frac{\mu \Phi }{\text{K}\left(1+\lambda_{1}\right)}\left(1+\lambda_{2}\frac{\text{d}}{\text{dt}}\right)v,$$ (10)

$$\rho \left[\frac{dw}{dt}\right]=-\frac{\partial p}{\partial z}+\frac{\partial S_{zx}}{\partial x}+\frac{\partial S_{zy}}{\partial y}+\frac{\partial S_{zz}}{\partial z}-\frac{\mu \Phi }{\text{K}\left(1+\lambda_{1}\right)}\left(1+\lambda_{2}\frac{\text{d}}{\text{dt}}\right)w.$$ (11)

in which the modified pressure $\hat{p}=p-\frac{1}{2}{\rho \Omega }^{2}\left(x^{2}+y^{2}\right).$ Employing Rosseland’s' approximation for radiative heat flux one has [19–20] $q_{r}=\frac{4\sigma^{*}}{3k^{*}}\frac{\partial T^{4}}{\partial z},$ where σ* and k* denote the Stefan-Boltzmann and Rosseland’s mean absorption coefficients respectively. We assume the temperature variations in such a way that Taylor's' series expansion of *T*^*4*^ about *T*_*m*_ (mean temperature) can be obtained. Neglecting higher order terms we get [40–41]

$${\text{T}}^{4}\approx 4{\text{T}}_{\text{m}}^{3}\text{T}-3{\text{T}}_{\text{m}}^{4}.$$ (12)

Through Eqs (4), (5) and (12) we arrive at

$$\begin{array}{l} \rho {\text{C}}_{\text{p}}\frac{\text{dT}}{\text{dt}}=\kappa \left[\frac{\partial^{2}\text{T}}{\partial {\text{x}}^{2}}+\frac{\partial^{2}\text{T}}{\partial {\text{y}}^{2}}+\frac{\partial^{2}\text{T}}{\partial {\text{z}}^{2}}\right]+{\text{S}}_{\text{xx}}\frac{\partial \text{u}}{\partial \text{x}}+{\text{S}}_{\text{xz}}\left(\frac{\partial \text{u}}{\partial \text{z}}+\frac{\partial \text{w}}{\partial \text{x}}\right)+{\text{S}}_{\text{zz}}\frac{\partial \text{w}}{\partial \text{z}}\\ +\frac{{\text{DK}}_{\text{r}}}{{\text{C}}_{\text{s}}}\left[\frac{\partial^{2}\text{C}}{\partial {\text{x}}^{2}}+\frac{\partial^{2}\text{C}}{\partial {\text{y}}^{2}}+\frac{\partial^{2}\text{C}}{\partial {\text{z}}^{2}}\right]+\frac{16\sigma^{\text{*}}{\text{T}}_{\text{m}}^{3}}{3{\text{k}}^{\text{*}}}\frac{\partial^{2}\text{T}}{\partial {\text{z}}^{2}}, \end{array}$$ (13)

$$\rho C_{p}\frac{dC}{dt}=D\left[\frac{\partial^{2}C}{\partial x^{2}}+\frac{\partial^{2}C}{\partial y^{2}}+\frac{\partial^{2}C}{\partial z^{2}}\right]+\frac{DK_{T}}{T_{m}}\left[\frac{\partial^{2}T}{\partial x^{2}}+\frac{\partial^{2}T}{\partial y^{2}}+\frac{\partial^{2}T}{\partial z^{2}}\right].$$ (14)

Equation of motion for the compliant walls can be expressed in the form

$$\text{L}\left(\eta \right)=\text{p}-{\text{p}}_{0}$$ (15)

with

$$\text{L}=-\tau^{\prime }\frac{\partial^{2}}{\partial x^{2}}+\text{m}\frac{\partial^{2}}{\partial t^{2}}+d^{\prime }\frac{\partial }{\partial t}+\text{b}\frac{\partial^{4}}{\partial x^{4}}+k_{2},$$ (16)

$$\begin{array}{l} \frac{\partial }{\partial x}L\left(\eta \right)=\frac{\partial p}{\partial x}=\frac{\partial S_{xx}}{\partial x}+\frac{\partial S_{xy}}{\partial y}+\frac{\partial S_{xz}}{\partial z}-\sigma B_{0}^{2}u+2\rho \Omega v-\rho \frac{du}{dt}-\frac{\mu \Phi }{K\left(1+\lambda_{1}\right)}\left(1+\lambda_{2}\frac{d}{dt}\right)u\\ at\text{ z}=\pm \eta . \end{array}$$ (17)

Non-dimensional variables can be put into the following forms:

$$x^{*}=\frac{x}{\lambda },\;\;y^{*}=\frac{y}{\lambda },\;z^{*}=\frac{z}{d},\;\;p^{*}=\frac{d^{2}p}{c\mu \lambda },\;t^{*}=\frac{ct}{\lambda },\;u^{*}=\frac{u}{c},\;\;v^{*}=\frac{v}{c},\;\;w^{*}=\frac{w}{c},\;{\text{S}}^{*}=\frac{\text{dS}}{\mu c},\;\eta^{*}=\frac{\eta }{d}$$

$$\lambda_{2}^{*}=\frac{c}{d}\lambda_{2},\;\theta =\frac{T-T_{0}}{T_{1}-T_{0}},\;\phi =\frac{C-C_{0}}{C_{1}-C_{0}}\;.$$

Using above mentioned variables and defining stream function by

$$\mu =\psi_{z},w=-\delta \psi_{x},$$

Eqs (9–11), (13) and (14) give

$$\begin{array}{l} \text{Re}\delta \left[\frac{\partial^{2}\psi }{\partial \text{z}\partial \text{t}}+\frac{\partial \psi }{\partial \text{z}}\frac{\partial^{2}\psi }{\partial \text{z}\partial \text{x}}-\frac{\partial \psi }{\partial \text{x}}\frac{\partial^{2}\psi }{\partial {\text{z}}^{2}}\right]-2\text{T}\prime \text{v}=-\frac{\partial \text{p}}{\partial \text{x}}+\delta \frac{\partial }{\partial \text{x}}{\text{S}}_{\text{xx}}+\delta \frac{\partial }{\partial \text{y}}{\text{S}}_{\text{xy}}+\frac{\partial }{\partial \text{z}}{\text{S}}_{\text{xz}}-{\text{M}}^{2}\psi_{\text{z}}\\ -\frac{1}{K_{1}\left(1+\lambda_{1}\right)}\left(1+\delta \lambda_{2}\frac{d}{dt}\right)\psi_{\text{z}}, \end{array}$$ (18)

$$\begin{array}{l} \text{Re}\delta \left[\frac{\partial \text{v}}{\partial \text{t}}+\frac{\partial \psi }{\partial \text{z}}\frac{\partial \text{v}}{\partial \text{x}}-\frac{\partial \psi }{\partial \text{x}}\frac{\partial \text{v}}{\partial \text{z}}\right]+2\text{T}\prime \frac{\partial \psi }{\partial \text{z}}=-\frac{\partial \text{p}}{\partial \text{y}}+\delta \frac{\partial }{\partial \text{x}}S_{\text{xy}}+\delta \frac{\partial }{\partial \text{y}}{\text{S}}_{\text{yy}}+\frac{\partial }{\partial \text{z}}{\text{S}}_{\text{yz}}-{\text{M}}^{2}v\\ -\frac{1}{K_{1}\left(1+\lambda_{1}\right)}\left(1+\delta \lambda_{2}\frac{d}{dt}\right)v, \end{array}$$ (19)

$$\begin{array}{l} Re\delta^{2}\left[-\frac{\partial^{2}\psi }{\partial x\partial t}-\frac{\partial \psi }{\partial z}\frac{\partial^{2}\psi }{\partial x^{2}}+\frac{\partial \psi }{\partial x}\frac{\partial^{2}\psi }{\partial x\partial z}\right]=-\frac{\partial p}{\partial z}+\delta \frac{\partial }{\partial x}S_{zx}+\delta \frac{\partial }{\partial y}S_{zy}+\frac{\partial }{\partial z}S_{zz}\\ +\frac{\delta }{K_{1}\left(1+\lambda_{1}\right)}\left(1+\delta \lambda_{2}\frac{d}{dt}\right)\psi_{x}, \end{array}$$ (20)

$$\begin{array}{l} \delta PrRe\left[\frac{\partial \theta }{\partial t}+\frac{\partial \psi }{\partial z}\frac{\partial \theta }{\partial x}+v\frac{\partial \theta }{\partial y}-\frac{\partial \psi }{\partial x}\frac{\partial \theta }{\partial z}\right]=\left[\delta^{2}\frac{\partial^{2}\theta }{\partial x^{2}}+\delta^{2}\frac{\partial^{2}\theta }{\partial y^{2}}+\frac{\partial^{2}\theta }{\partial z^{2}}\right]+R\delta^{2}\frac{\partial^{2}\theta }{\partial x^{2}}\\ +DuPr\left[\delta^{2}\frac{\partial^{2}\phi }{\partial x^{2}}+\delta^{2}\frac{\partial^{2}\phi }{\partial y^{2}}+\frac{\partial^{2}\phi }{\partial z^{2}}\right]+\frac{EcPr}{\left(1+\lambda_{1}\right)}\\ \times \left(1+\delta \lambda_{2}\frac{d}{dt}\right)\left[4\delta^{2}{\left(\psi_{zz}\right)}^{2}+{\left(\psi_{zz}-\delta^{2}\psi_{xx}\right)}^{2}\right], \end{array}$$ (21)

$$\begin{array}{l} Re\delta \left[\frac{\partial \phi }{\partial t}+\frac{\partial \psi }{\partial z}\frac{\partial \phi }{\partial x}+v\frac{\partial \phi }{\partial y}-\frac{\partial \psi }{\partial x}\frac{\partial \phi }{\partial z}\right]=\frac{1}{Sc}\left[\delta^{2}\frac{\partial^{2}\phi }{\partial x^{2}}+\delta^{2}\frac{\partial^{2}\phi }{\partial y^{2}}+\frac{\partial^{2}\phi }{\partial z^{2}}\right]\\ +Sr\left[\delta^{2}\frac{\partial^{2}\theta }{\partial x^{2}}+\delta^{2}\frac{\partial^{2}\theta }{\partial y^{2}}+\frac{\partial^{2}\theta }{\partial z^{2}}\right]. \end{array}$$ (22)

Where continuity Eq (8) is identically satisfied. The boundary conditions are now reduced as follows:

$$\psi_{z}=0,v=0,\theta =\left(\begin{array}{c} 1\\ 0 \end{array}\right),\;\phi =\left(\begin{array}{c} 1\\ 0 \end{array}\right)\;\;at\;\;z=\pm \eta ,$$ (23)

$$\begin{array}{l} \left[E_{1}\frac{\partial^{3}}{\partial x^{3}}+E_{2}\frac{\partial^{3}}{\partial x\partial t^{2}}+E_{3}\frac{\partial^{2}}{\partial x\partial t}+E_{4}\frac{\partial^{5}}{\partial x^{5}}+E_{5}\frac{\partial }{\partial x}\right]\eta =\delta \frac{\partial }{\partial x}S_{xx}+\delta \frac{\partial }{\partial y}S_{xy}+\delta \frac{\partial }{\partial z}S_{xz}\\ -M^{2}\frac{\partial \psi }{\partial z}+2T^{\prime }v-\delta \text{Re}\frac{d\psi_{z}}{dt}-\frac{1}{K_{1}\left(1+\lambda_{1}\right)}\left(1+\delta \lambda_{2}\frac{d}{dt}\right)\psi_{z}\;at\;z=\pm \eta \end{array}$$ (24)

η=1+εsin[2π(x−t)].

In above expressions Re *(= cd/ν)* is the Reynolds number, *δ (= a/d)* the wave number, *T*^*’*^ *(= ReΩd/c)* the Taylor number, *M*^*2*^ *(= B*_*0*_*d*^*2*^*σ/μ)* the Hartman number, *K*_*1*_ *(= K/Φd*^*2*^*)* the medium permeability parameter, *Pr (= μC*_*p*_*/κ)* the Prandtl number, R (= 16σ^*^ *T*^*3*^_*1*_/3kk*) the radiation parameter, *Ec (= c*^*2*^*/C*_*p*_*(T*_*1*_*-T*_*0*_*))* the Eckert number, *Du (= DK*_*T*_*(C*_*1*_*-C*_*0*_*)/C*_*s*_*C*_*p*_*μ(T*_*1*_*-T*_*0*_*))* the Dufour number, *Sr (= ρDK*_*T*_*(T*_*1*_*-T*_*0*_*)/μT*_*m*_*(C*_*1*_*-C*_*0*_*))* the Soret number, *Sc (= ν/D)* the Schmidt number, *ε (= a/d)* the amplitude ratio and *E*_*1*_ *(= -τd*^*3*^*/λ*^*3*^*μc)*, *E*_*2*_ *(= m*_*1*_*cd*^*3*^*/λ*^*3*^*μ)*, *E*_*3*_ *(= d*^*’*^*d/λ*^*2*^*μ)*, *E*_*4*_ *(= bd*^*3*^*/ λ*^*5*^*μc)* and *E*_*1*_ *(= k*_*2*_ *d*^*3*^*/λμc)* the non-dimensional elasticity parameters. Invoking long wavelength and low Reynolds number approximations we obtain

$$-2T^{\prime }v=-\frac{\partial p}{\partial x}+\frac{1}{\left(1+\lambda_{1}\right)}\psi_{zzz}-\left(M^{2}+\frac{1}{K_{1}\left(1+\lambda_{1}\right)}\right)\psi_{z},$$ (25)

$$2T^{\prime }\psi_{z}=-\frac{\partial p}{\partial y}+\frac{1}{\left(1+\lambda_{1}\right)}v_{zz}-\left(M^{2}+\frac{1}{K_{1}\left(1+\lambda_{1}\right)}\right)v,$$ (26)

$$\frac{\partial p}{\partial z}=0,$$ (27)

$$\theta_{zz}=-\frac{EcPr}{\left(1+R\right)\left(1+\lambda_{1}\right)}\psi_{zz}^{2}-\frac{DuPr}{\left(1+R\right)}\phi_{zz},$$ (28)

$$\phi_{zz}+{\text{ScSr}\theta }_{zz}=0,$$ (29)

$$\psi_{z}=0,v=0,\theta =\left(\begin{array}{c} 1\\ 0 \end{array}\right),\;\phi =\left(\begin{array}{c} 1\\ 0 \end{array}\right)\text{ at z}=\pm \eta ,$$ (30)

$$\begin{array}{l} \left[E_{1}\frac{\partial^{3}}{\partial x^{3}}+E_{2}\frac{\partial^{3}}{\partial x\partial t^{2}}+E_{3}\frac{\partial^{2}}{\partial x\partial t}+E_{4}\frac{\partial^{5}}{\partial x^{5}}+E_{5}\frac{\partial }{\partial x}\right]\eta =\\ \left[E_{1}\frac{\partial^{3}}{\partial x^{3}}+E_{2}\frac{\partial^{3}}{\partial x\partial t^{2}}+E_{3}\frac{\partial^{2}}{\partial x\partial t}+E_{4}\frac{\partial^{5}}{\partial x^{5}}+E_{5}\frac{\partial }{\partial x}\right]\eta \\ =\frac{1}{\left(1+\lambda_{1}\right)}\psi_{zzz}-\left(M^{2}+\frac{1}{K_{1}\left(1+\lambda_{1}\right)}\right)\psi_{z}+2T^{\prime }v\;\;at\;\;z=\pm \eta . \end{array}$$ (31)

Eq (27) shows that pressure is not a function of *z*. Hence, the pressure can be eliminated from Eq (25). Further, pressure term in Eq (26) can be neglected since the secondary flow is resulted by the rotation [36]. In view of these facts we can write Eqs (25) and (26) in the forms

$$-2T^{\prime }v_{z}=\frac{1}{\left(1+\lambda_{1}\right)}\psi_{zzz}-\left(M^{2}+\frac{1}{K_{1}\left(1+\lambda_{1}\right)}\right)\psi_{zz},$$ (32)

$$2T^{\prime }\psi_{z}=\frac{1}{\left(1+\lambda_{1}\right)}v_{zz}-\left(M^{2}+\frac{1}{K_{1}\left(1+\lambda_{1}\right)}\right)v.$$ (33)

### Exact solutions

Solving Eqs (32) and (33) we have the following relations of stream function and secondary velocity

$$\psi =B_{13}\text{z}+B_{14}\text{sinh}\left[\sqrt{B_{1}}\text{z}\right]+B_{15}\text{sinh}\left[\sqrt{B_{2}}\text{z}\right],$$ (34)

$$\begin{array}{l} v=\left(B_{21}+B_{22}\text{cosh}\left[\sqrt{B_{1}z}\right]+B_{23}\text{cosh}\left[\sqrt{B_{2}z}\right]\right)\times (cosh^{2}\left[\left(\sqrt{B_{1}}+\sqrt{B_{2}}\right)z\right]-sinh^{2}[(\sqrt{B_{1}}\\ +\sqrt{B_{2}})z]). \end{array}$$ (35)

Making use of Eqs (34) and (35) into Eqs (28) and (29) and solving the resulting expressions through lubrication approach we have

$$\begin{array}{l} \theta =A_{3}+A_{4}\text{z}+A_{5}z^{2}+A_{6}\text{cosh}\left[2\sqrt{B_{1}}\text{z}\right]+A_{7}\text{cosh}\left[2\sqrt{B_{2}}\text{z}\right]+A_{8}\text{cosh}\left[2\sqrt{B_{2}}\text{z}-2\sqrt{B_{1}}\eta \right]\\ +A_{9}\text{cosh}\left[2\sqrt{B_{2}}z+2\sqrt{B_{1}}\eta \right]+A_{10}\text{cosh}\left[2\sqrt{B_{1}}z-2\sqrt{B_{2}}\eta \right]+A_{11}\text{cosh}\left[2\sqrt{B_{1}}z+2\sqrt{B_{2}}\eta \right]\\ -A_{12}\text{cosh}\left[\left(\sqrt{B_{1}}+\sqrt{B_{2}}\right)z-\left(\sqrt{B_{1}}+\sqrt{B_{2}}\right)\eta \right]+A_{13}\text{cosh}\left[\left(\sqrt{B_{1}}-\sqrt{B_{2}}\right)z\right]\\ +A_{14}\text{cosh}\left[\left(\sqrt{B_{1}}+\sqrt{B_{2}}\right)z\right]-A_{15}\text{sinh}\left[\left(\sqrt{B_{1}}+\sqrt{B_{2}}\right)\eta \right], \end{array}$$ (36)

$$\begin{array}{l} \phi =F_{1}z+F_{2}z^{2}+F_{3}\text{cosh}\left[2\sqrt{B_{1}}z\right]+F_{4}\text{cosh}\left[2\sqrt{B_{2}}z\right]+F_{5}\text{cosh}{\left[2\sqrt{B_{1}}z\right]}^{2}\\ +F_{6}\text{cosh}\left[\sqrt{B_{1}}z\right]\text{cosh}\left[\sqrt{B_{2}}z\right]+F_{7}\text{sinh}{\left[\sqrt{B_{1}}z\right]}^{2}+F_{8}\text{sinh}{\left[\sqrt{B_{2}}z\right]}^{2}+F_{9}\text{sinh}\left[\sqrt{B_{1}}z\right]\text{sinh}\left[\sqrt{B_{2}}z\right]\\ +F_{10}. \end{array}$$ (37)

With

$${\text{B}}_{1}=\alpha -2i\beta ,$$

$${\text{B}}_{2}=\alpha -2i\beta ,$$

$$\alpha =\left(M^{2}+\frac{1}{K_{1}\left(1+\lambda_{1}\right)}\right)\left(1+\lambda_{1}\right),$$

$$\beta =\text{T}\prime (1+\lambda_{1}).$$

Heat transfer coefficient at the wall is given below

$$\begin{array}{l} Z=\eta_{x}\theta \left(\eta \right),\\ =\eta_{x}[A_{4}+2A_{5}\eta +2\sqrt{B_{1}}A_{6}\text{sinh}\left[2\sqrt{B_{1}}\eta \right]+2\sqrt{B_{2}}A_{7}\text{sinh}\left[2\sqrt{B_{2}}\eta \right]\\ +2\sqrt{B_{2}}A_{8}\text{sinh}\left[2\left(\sqrt{B_{2}}-\sqrt{B_{1}}\right)\eta \right]+2\sqrt{B_{2}}A_{9}\text{sinh}\left[2\left(\sqrt{B_{2}}+\sqrt{B_{1}}\right)\eta \right]\\ +2\sqrt{B_{1}}A_{10}\text{sinh}\left[2\left(\sqrt{B_{1}}-\sqrt{B_{2}}\right)\eta \right]+2\sqrt{B_{1}}A_{11}\text{sinh}\left[2\left(\sqrt{B_{1}}+\sqrt{B_{2}}\right)\eta \right]\\ +\left(\sqrt{B_{1}}-\sqrt{B_{2}}\right)A_{13}\text{sinh}\left[\left(\sqrt{B_{1}}-\sqrt{B_{2}}\right)\eta \right]+\left(\sqrt{B_{1}}+\sqrt{B_{2}}\right)A_{14}\text{sinh}\left[\left(\sqrt{B_{1}}+\sqrt{B_{2}}\right)\eta \right]], \end{array}$$ (38)

in which *A*_4_ → *A*_15_, *B*_13_→ *B*_23_, *F*_1_ →*F*_10_ can be calculated algebraically.

## Discussion

**Fig 2:**
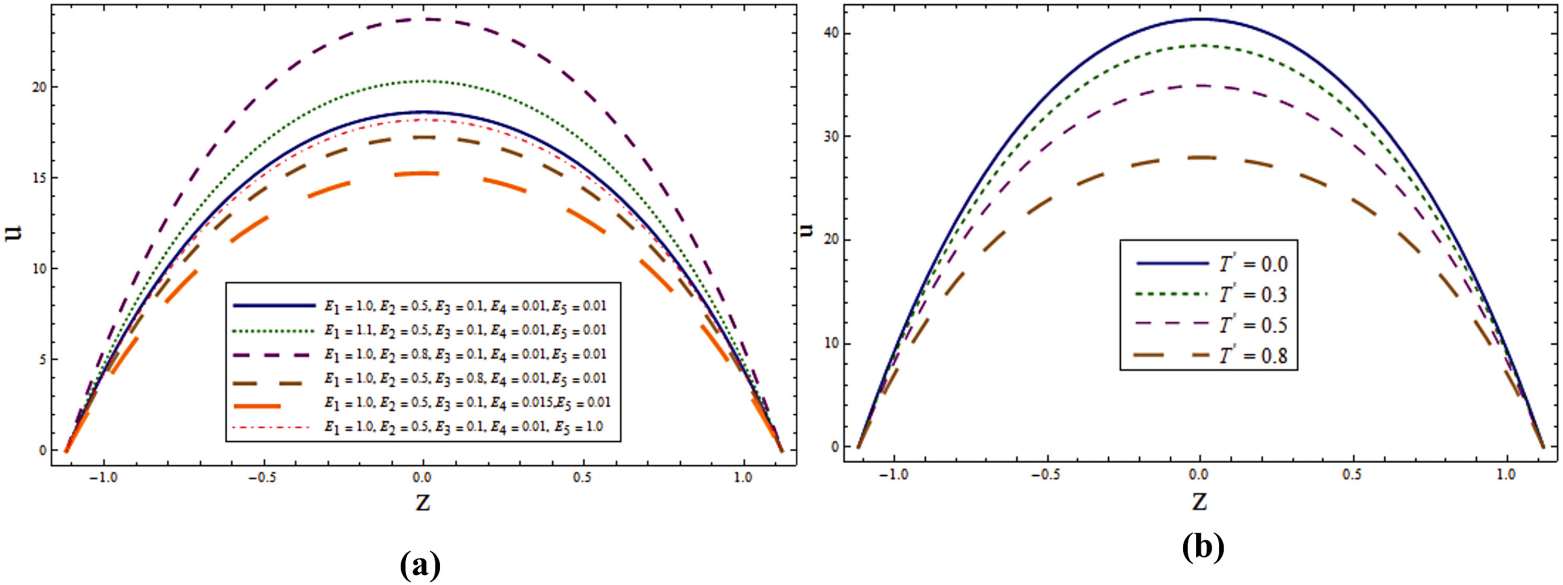
**(a)** Impact of wall properties on **u** when *T*^*'*^ = *λ*_*1*_ = *M = K*_*1*_ = *0*.*5*, *x = ε = 0*.*2* and *t = 0*.*1*. **(b)** Impact of ***T***^***'***^ on **u** when *E*_*1*_ = *E*_*2*_ = *3*.*0*, *E*_*3*_ = *0*.*01*, *E*_*4*_ = *E*_*5*_ = *0*.*1*, *λ*_*1*_ = *M = K*_*1*_ = *0*.*5*, *x = ε = 0*.*2* and *t = 0*.*1*.

**Fig 3:**
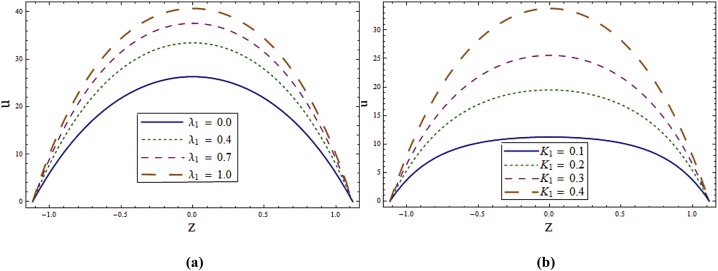
**(a)** Impact of ***λ***_***1***_ on **u** when *E*_*1*_ = *E*_*2*_ = *3*.*0*, *E*_*3*_ = *0*.*01*, *E*_*4*_ = *E*_*5*_ = *0*.*1*, *T*^*'*^ = *λ*_*1*_ = *M = K*_*1*_ = *0*.*5*, *x = ε = 0*.*2* and *t = 0*.*1*. **(b)** Impact of ***K***_***1***_ on **u** when *E*_*1*_ = *E*_*2*_ = *3*.*0*, *E*_*3*_ = *0*.*01*, *E*_*4*_ = *E*_*5*_ = *0*.*1*, *T*^*'*^ = *λ*_*1*_ = *M = 0*.*5*, *x = ε = 0*.*2* and *t = 0*.*1*.

**Fig 4:**
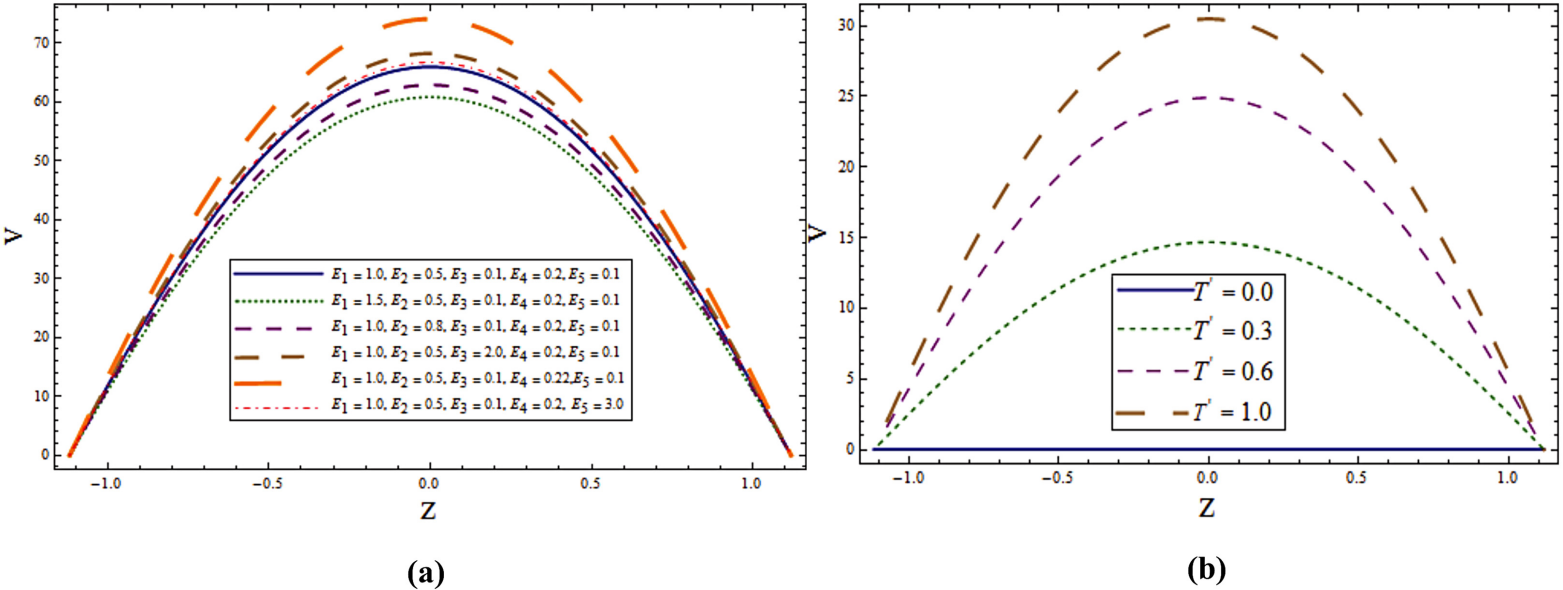
**(a)** Impact of wall properties on **v** when *T*^*'*^ = *λ*_*1*_ = *M = K*_*1*_ = *0*.*5*, *x = ε = 0*.*2* and *t = 0*.*1*. **(b)** Impact of ***T***^***'***^ on **v** when *E*_*1*_ = *E*_*2*_ = *3*.*0*, *E*_*3*_ = *0*.*01*, *E*_*4*_ = *E*_*5*_ = *0*.*1*, *λ*_*1*_ = *M = K*_*1*_ = *0*.*5*, *x = ε = 0*.*2* and *t = 0*.*1*.

**Fig 5:**
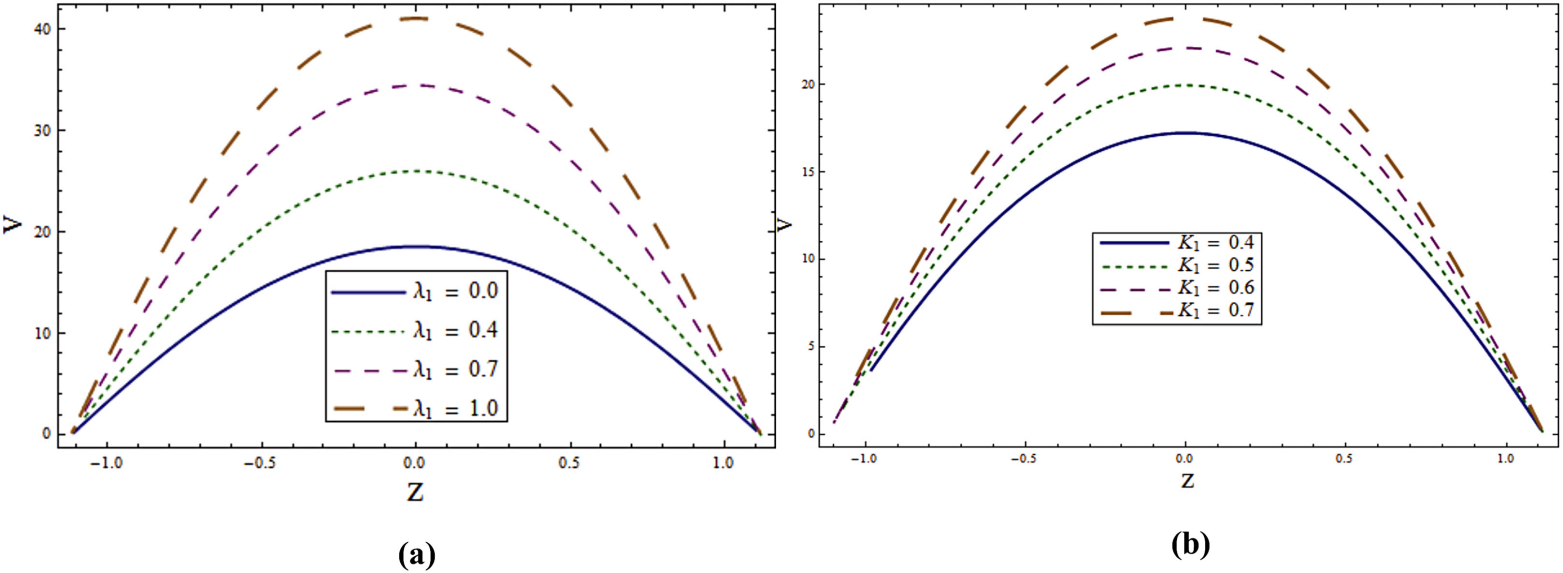
**(a)** Impact of ***λ***_***1***_ on **v** when *E*_*1*_ = *E*_*2*_ = *3*.*0*, *E*_*3*_ = *0*.*01*, *E*_*4*_ = *E*_*5*_ = *0*.*1*, *T*^*'*^ = *λ*_*1*_ = *M = K*_*1*_ = *0*.*5*, *x = ε = 0*.*2* and *t = 0*.*1*. **(b)** Impact of ***K***_***1***_ on **v** when *E*_*1*_ = *E*_*2*_ = *3*.*0*, *E*_*3*_ = *0*.*01*, *E*_*4*_ = *E*_*5*_ = *0*.*1*, *T*^*'*^ = *λ*_*1*_ = *M = 0*.*5*, *x = ε = 0*.*2* and *t = 0*.*1*.

The main objective here is to predict the impact of sundry parameters on the velocity, temperature and concentration profiles. The theme of present study is to analyze the influence of rotation in the presence of Soret and Dufour effects. Here Figs 2 and 3 are prepared for the velocity and temperature whereas the Figs 4 and 5 show the variations of concentration and heat transfer rate respectively.

Here Figs 2 and 3 are prepared to analyze the axial velocity. These Figures show that velocity traces a parabolic path with maximum value at the center of channel. Fig 2a shows that velocity enhances when elasticity parameters *E*_*1*_ and *E*_*2*_ are increased. There is decrease in velocity for larger *E*_*3*_, *E*_*4*_ and *E*_*5*_. Since *E*_*1*_ and *E*_*2*_ represent the elastic parameters therefore increasing elasticity offers less resistance to the flow and hence velocity increases. On the contrary the wall damping creates a resistive type force and so the velocity decreases when *E*_*3*_ increases. Similar behavior is noticed for *E*_*4*_ and *E*_*5*_ in the presence of damping. Fig 2b illustrates that increasing *T*^*'*^ reduces the velocity of the fluid in axial direction. This Figure also provides a comparison for axial velocity for rotating and nonrotating channels. It is found that axial velocity is greater in non-rotating channel i.e. (*T = 0*^*'*^). Fig 3a shows an increase in velocity when *λ*_*1*_ is increased. Velocity enhances when the value of *K*_*1*_ is increased (see Fig 3b). Porosity parameter depends on the permeability parameter *K*. Increase *K*_*1*_ in leads to the higher permeability parameter. Ultimately the velocity thus increases through larger *K*_*1*_.

Figs 4 and 5 highlight the effects of wall properties, *T*^*'*^, *λ*_*1*_ and *K*_*1*_ on the secondary velocity. The rotation of channel induces a velocity component in the *y*- direction which in turn produces a fluid flow in *y*- direction which is termed as secondary flow. We can observe the effect of wall properties on secondary flow ν through Fig 4a. Decrease in ν is observed by increasing *E*_*1*_ and *E*_*2*_ while it enhances through *E*_*3*_, *E*_*4*_ and *E*_*5*_. In the absence of rotation there is no secondary velocity but velocity in *y*- direction increases in presence of rotation (see Fig 4b). Fig 5a shows that the secondary velocity ν increases for larger *λ*_*1*_. Similar effect is shown by *K*_*1*_ which is observed in Fig 5b.

**Fig 6:**
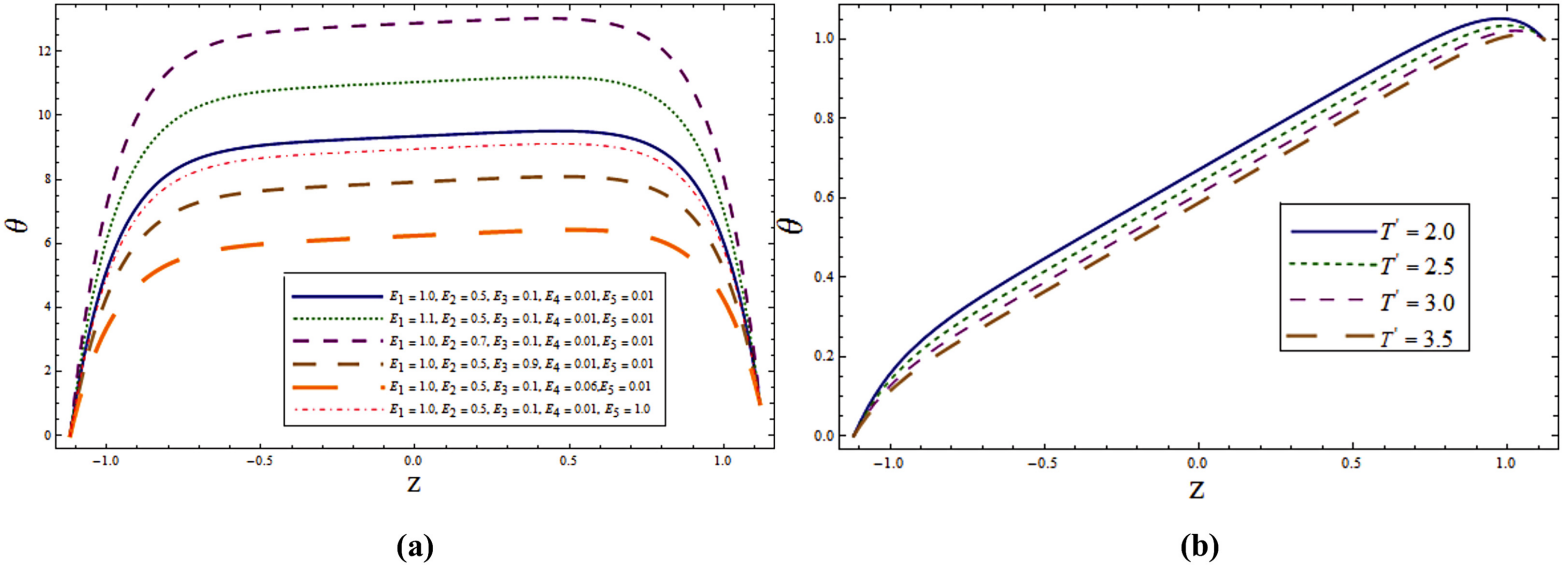
**(a)** Variation of wall properties on **θ** when *Sr = Du = Sc = R = λ*_*1*_ = *M = 0*.*8*, *T*^*'*^ = *Pr = 1*.*0*, *K*_*1*_ = *0*.*1*, *Ec = 2*.*0*, *x = ε = 0*.*2* and *t = 0*.*1*. **(b)** Variation of ***T***^***'***^ on **θ** when *E*_*1*_ = *0*.*2*, *E*_*2*_ = *E*_*3*_ = *E*_*4*_ = *0*.*01*, *E*_*5*_ = *0*.*1*, *Sr = Du = Sc = R = λ*_*1*_ = *M = 0*.*8*, *K*_*1*_ = *0*.*1*, *Ec = 2*.*0*, Pr = 1.0, *x = ε = 0*.*2* and *t = 0*.*1*.

**Fig 7:**
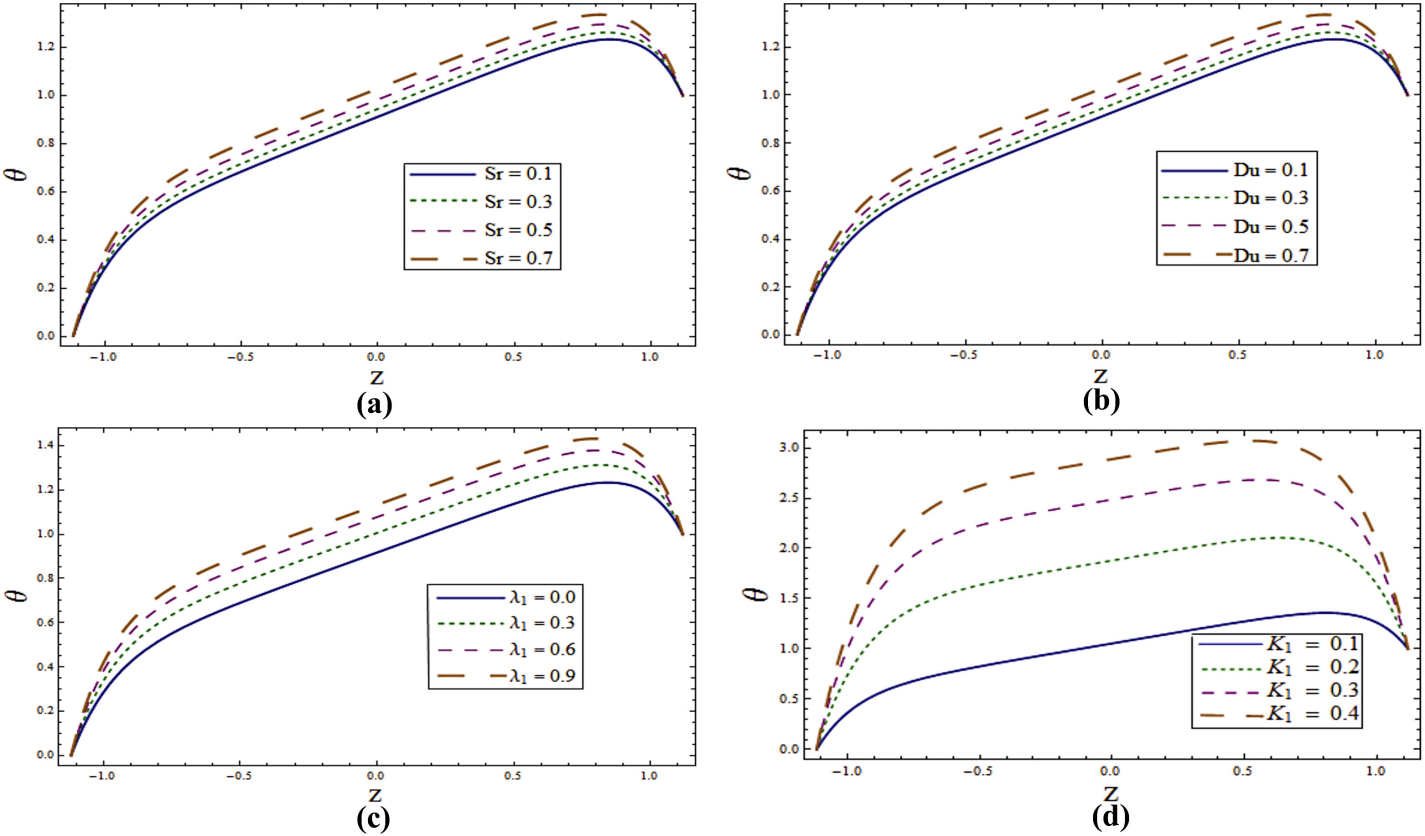
**(a)** Variation of **Sr** on **θ** when *E*_*1*_ = *0*.*2*, *E*_*2*_ = *E*_*3*_ = *E*_*4*_ = *0*.*01*, *E*_*5*_ = *0*.*1*, *Du = Sc = R = λ*_*1*_ = *M = 0*.*8*, *K*_*1*_ = *0*.*1*, *Ec = 2*.*0*, *T'* = Pr = 1.0, *x = ε = 0*.*2* and *t = 0*.*1*. **(b)** Variation of ***Du*** on **θ** when *E*_*1*_ = *0*.*2*, *E*_*2*_ = *E*_*3*_ = *E*_*4*_ = *0*.*01*, *E*_*5*_ = *0*.*1*, *Sr = Sc = R = λ*_*1*_ = *M = 0*.*8*, *K*_*1*_ = *0*.*1*, *Ec = 2*.*0*, *T'* = Pr = 1.0, *x = ε = 0*.*2* and *t = 0*.*1*. **(c)** Variation of ***λ***_***1***_ on **θ** when *E*_*1*_ = *0*.*2*, *E*_*2*_ = *E*_*3*_ = *E*_*4*_ = *0*.*01*, *E*_*5*_ = *0*.*1*, *Sr = Sc = R = Du = M = 0*.*8*, *K*_*1*_ = *0*.*1*, *Ec = 2*.*0*, *T'* = Pr = 1.0, *x = ε = 0*.*2* and *t = 0*.*1*. **(d)** Variation of ***K***_***1***_ on **θ** when *E*_*1*_ = *0*.*2*, *E*_*2*_ = *E*_*3*_ = *E*_*4*_ = *0*.*01*, *E*_*5*_ = *0*.*1*, *Sr = Sc = R = Du = λ*_*1*_ = *M = 0*.*8*, *Ec = 2*.*0*, *T'* = Pr = 1.0, *x = ε = 0*.*2* and *t = 0*.*1*.

**Fig 8:**
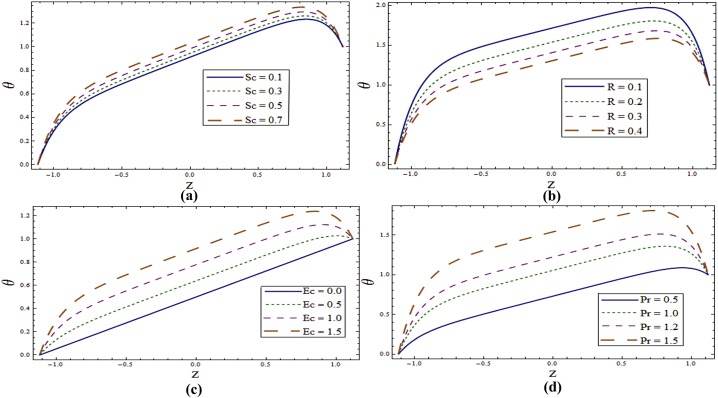
**(a)** Variation of **Sc** on **θ** when *E*_*1*_ = *0*.*2*, *E*_*2*_ = *E*_*3*_ = *E*_*4*_ = *0*.*01*, *E*_*5*_ = *0*.*1*, *Sr = Du = R = λ*_*1*_ = *M = 0*.*8*, *K*_*1*_ = *0*.*1*, *Ec = 2*.*0*, *T'* = Pr = 1.0, *x = ε = 0*.*2* and *t = 0*.*1*. **(b)** Variation of ***R*** on **θ** when *E*_*1*_ = *0*.*2*, *E*_*2*_ = *E*_*3*_ = *E*_*4*_ = *0*.*01*, *E*_*5*_ = *0*.*1*, *Sr = Du = Sc = λ*_*1*_ = *M = 0*.*8*, *K*_*1*_ = *0*.*1*, *Ec = 2*.*0*, *T'* = Pr = 1.0, *x = ε = 0*.*2* and *t = 0*.*1*. **(c)** Variation of ***Ec*** on **θ** when *E*_*1*_ = *0*.*2*, *E*_*2*_ = *E*_*3*_ = *E*_*4*_ = *0*.*01*, *E*_*5*_ = *0*.*1*, *Sr = Du = Sc = R = λ*_*1*_ = *M = 0*.*8*, *K*_*1*_ = *0*.*1*, *T'* = Pr = 1.0, *x = ε = 0*.*2* and *t = 0*.*1*. **(d)** Variation of **Pr** on **θ** when *E*_*1*_ = *0*.*2*, *E*_*2*_ = *E*_*3*_ = *E*_*4*_ = *0*.*01*, *E*_*5*_ = *0*.*1*, *Sr = Du = Sc = R = λ*_*1*_ = *M = 0*.*8*, *K*_*1*_ = *0*.*1*, *Ec = 2*.*0*, *T'* = 1.0, *x = ε = 0*.*2* and *t = 0*.*1*.

Impact of different parameters on temperature profile can be seen from Figs 6–8. It is a known fact that temperature is the average kinetic energy of particles which in turn depends on the velocity. Therefore an increase in temperature θ is noticed for increasing values of *E*_*1*_ and *E*_*2*_. On the contrary decrease in temperature is noticed for increasing values of *E*_*3*_, *E*_*4*_ and *E*_*5*_ (see Fig 6a). Fig 6b reveals that an increase in *T*^*'*^ causes decrease in θ. It is noticed that temperature enhances when we increase *Sr* and *Du*(see Fig 7a and 7b). In fact for increasing *Sr* and *Du* the thermal diffusion is increased and consequently the temperature enhances. Physically the diffusion-thermo or Dufour effect defines a heat flux produced when a chemical system undergoes a concentration gradient. These effects depend upon thermal diffusion which is though very small, but sometimes become substantial when the partaking species differ by molecular weights. Mass diffusion follows by the uneven distribution of species creating a concentration gradient. A temperature gradient can also work as a driving force for mass diffusion called thermo-diffusion or Soret effect. Therefore the higher the temperature gradient, the larger the Soret effect. Fig 7c reveals that temperature increases when the value of λ_1_ is increased. In fact higher λ_1_ corresponds to larger relaxation time which provides more resistance to the fluid motion and thus the temperature profile enhances. As by increasing the value of porosity parameter *K*_*1*_ the permeability of the medium increases which accelerates the fluid and thus temperature enhances (Fig 7d). Fig 8a and 8b display that temperature is decreasing function of *Sc* and *R*. Influence of Eckert number *Ec* on *θ* is displayed in Fig 8c. It is depicted from this Fig. that temperature enhances by increasing *Ec*. The heat generation due to internal friction caused by the shear in the flow is the reason behind such increase. Similar behavior is observed for Prandtl number *Pr* (Fig 8d).

**Fig 9:**
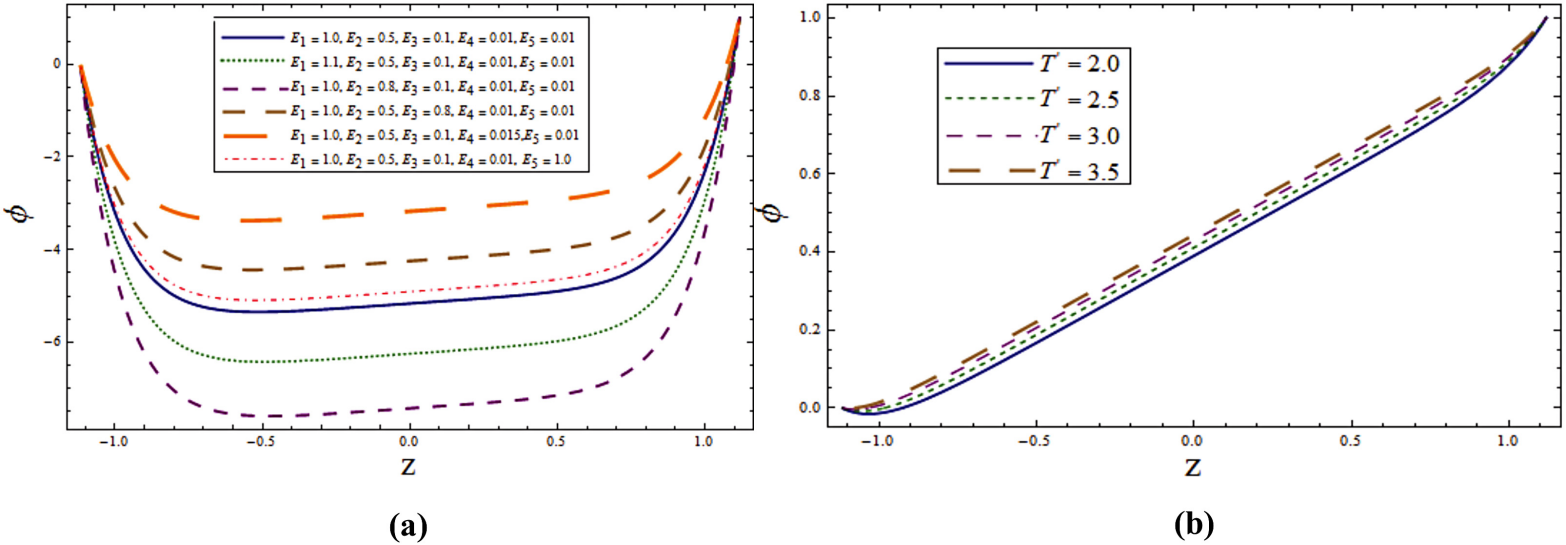
**(a)** Influence of wall properties on **ϕ** when *Sr = Du = Sc = R = λ*_*1*_ = *M = 0*.*8*, *K*_*1*_ = *0*.*1*, *Ec = 2*.*0*, *T' = Pr* = 1.0, *x = ε = 0*.*2* and *t = 0*.*1*. **(b)** Influence of ***T'*** on **ϕ** when *E*_*1*_ = *0*.*2*, *E*_*2*_ = *E*_*3*_ = *E*_*4*_ = *0*.*01*, *E*_*5*_ = *0*.*1*, *Sr = Du = Sc = R = λ*_*1*_ = *M = 0*.*8*, *K*_*1*_ = *0*.*1*, *Ec = 2*.*0*, *Pr* = 1.0, *x = ε = 0*.*2* and *t = 0*.*1*.

**Fig 10:**
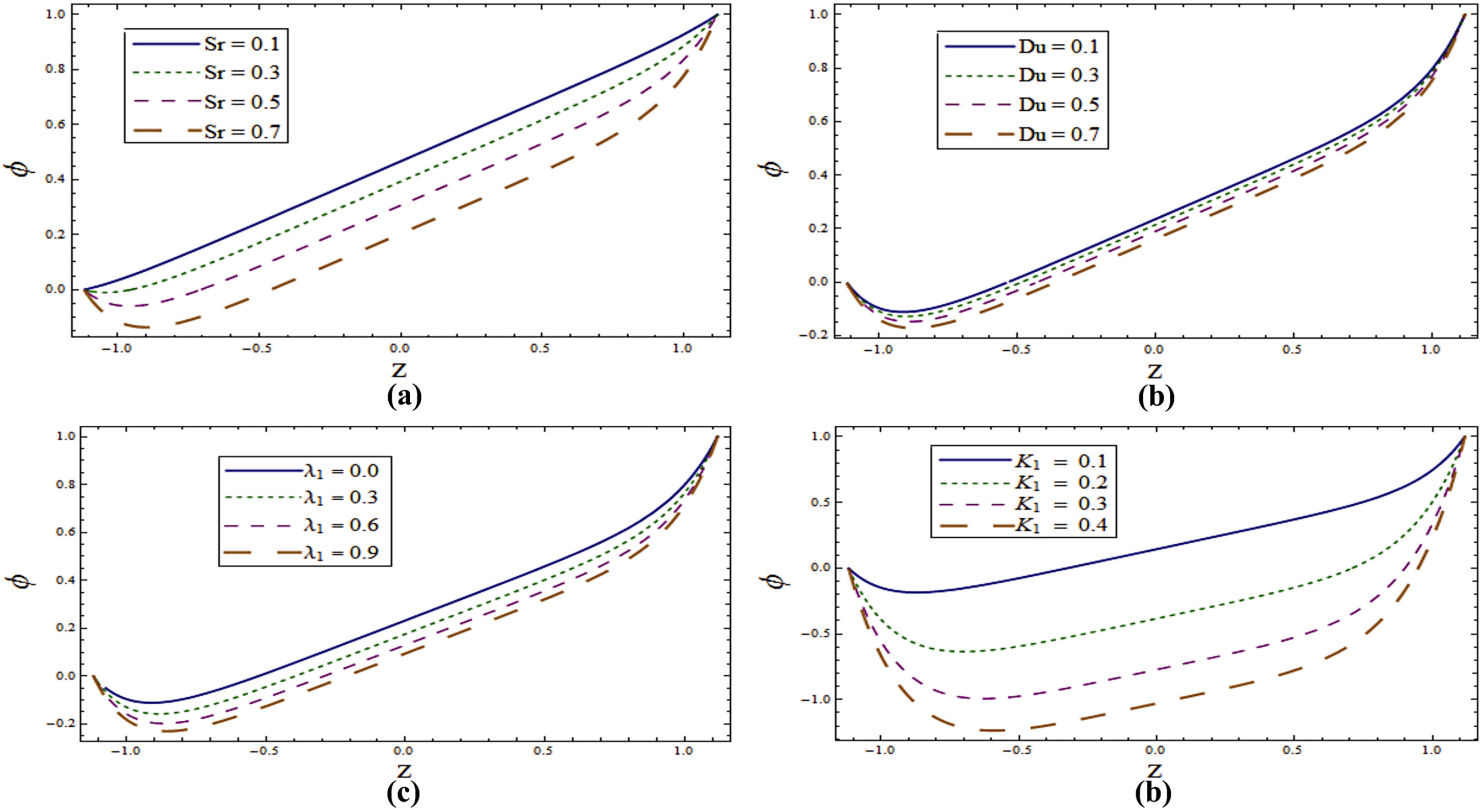
**(a)** Influence of **Sr** on **ϕ** when *E*_*1*_ = *0*.*2*, *E*_*2*_ = *E*_*3*_ = *E*_*4*_ = *0*.*01*, *E*_*5*_ = *0*.*1*, *Du = Sc = R = λ*_*1*_ = *M = 0*.*8*, *K*_*1*_ = *0*.*1*, *Ec = 2*.*0*, *T' = Pr* = 1.0, *x = ε = 0*.*2* and *t = 0*.*1*. **(b)** Influence of ***Du*** on **ϕ** when *E*_*1*_ = *0*.*2*, *E*_*2*_ = *E*_*3*_ = *E*_*4*_ = *0*.*01*, *E*_*5*_ = *0*.*1*, *Sr = Sc = R = λ*_*1*_ = *M = 0*.*8*, *K*_*1*_ = *0*.*1*, *Ec = 2*.*0*, *T' = Pr* = 1.0, *x = ε = 0*.*2* and *t = 0*.*1*. **(c)** Influence of ***λ***_***1***_ on **ϕ** when *E*_*1*_ = *0*.*2*, *E*_*2*_ = *E*_*3*_ = *E*_*4*_ = *0*.*01*, *E*_*5*_ = *0*.*1*, *Sr = Du = Sc = R = M = 0*.*8*, *K*_*1*_ = *0*.*1*, *Ec = 2*.*0*, *T' = Pr* = 1.0, *x = ε = 0*.*2* and *t = 0*.*1*. **(d)** Influence of ***K***_***1***_ on **ϕ** when *E*_*1*_ = *0*.*2*, *E*_*2*_ = *E*_*3*_ = *E*_*4*_ = *0*.*01*, *E*_*5*_ = *0*.*1*, *Sr = Du = Sc = R = λ*_*1*_ = *M = 0*.*8*, *Ec = 2*.*0*, *T' = Pr* = 1.0, *x = ε = 0*.*2* and *t = 0*.*1*.

**Fig 11:**
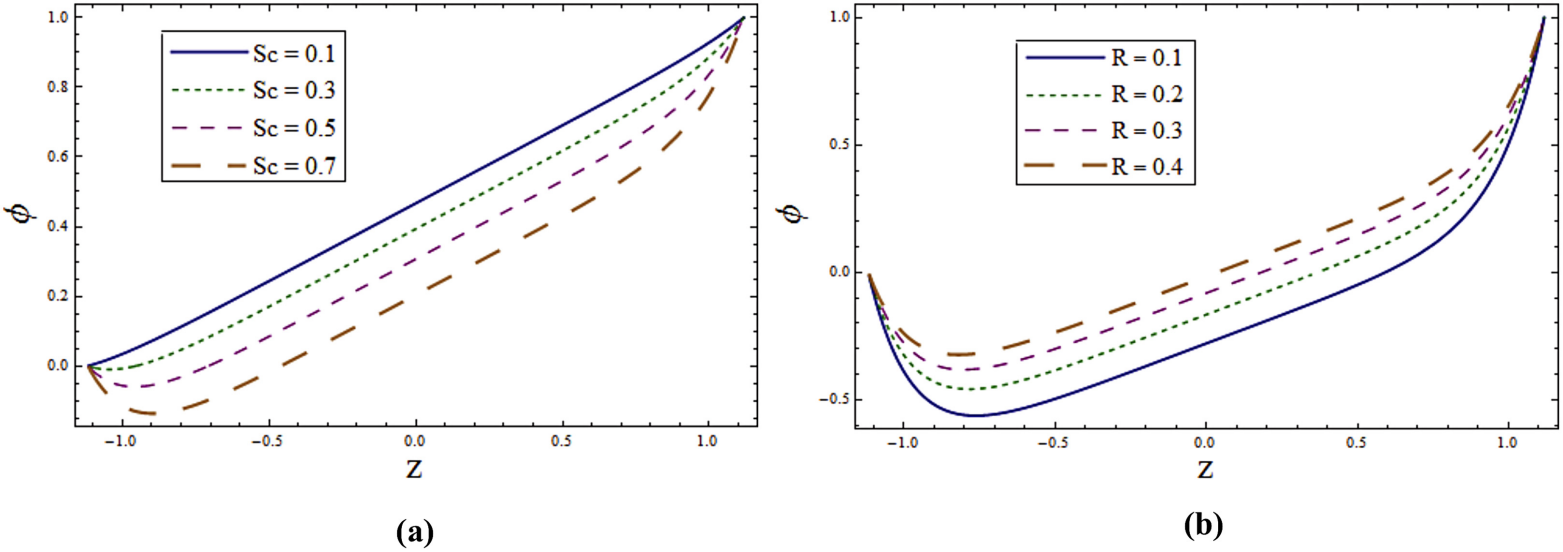
**(a)** Influence of **Sc** on **ϕ** when *E*_*1*_ = *0*.*2*, *E*_*2*_ = *E*_*3*_ = *E*_*4*_ = *0*.*01*, *E*_*5*_ = *0*.*1*, *Du = Sr = R = λ*_*1*_ = *M = 0*.*8*, *K*_*1*_ = *0*.*1*, *Ec = 2*.*0*, *T' = Pr* = 1.0, *x = ε = 0*.*2* and *t = 0*.*1*. **(b)** Influence of **R** on **ϕ** when *E*_*1*_ = *0*.*2*, *E*_*2*_ = *E*_*3*_ = *E*_*4*_ = *0*.*01*, *E*_*5*_ = *0*.*1*, *Du = Sr = Sc = λ*_*1*_ = *M = 0*.*8*, *K*_*1*_ = *0*.*1*, *Ec = 2*.*0*, *T' = Pr* = 1.0, *x = ε = 0*.*2* and *t = 0*.*1*.

Figs 9–11 disclose the effect of various parameters on concentration ϕ. Fig 9a displays the role of wall properties on *ϕ*. Decrease in concentration is noticed for increasing values of *E*_*1*_ and *E*_*2*_ but it increases for larger *E*_*3*,_ *E*_*4*_ and *E*_*5*._ Fig 9b examines the behavior of ϕ for Taylor number *T*^*'*^. It is revealed that *ϕ* is increasing function of Taylor number. Concentration distribution for various values of *Sr* and *Du* is displayed in Fig 10a and 10b. Here concentration decreases with the increase of these parameters. Fig 10c and 10d display against the various values of λ_1_ and *K*_*1*_. These Figures witness that *ϕ* decreases by increasing λ_1_ and *K*_*1*_. Fig 11a depicts that the concentration profile decreases when *Sc* increases. As Schmidt number is defined as the ratio of momentum diffusivity (viscosity) to mass diffusivity. Therefore increasing *Sc* decreases the mass diffusion which in turn reduces the concentration. For larger *R* concentration increases (see Fig 11b). It can be observed through the graphical results that concentration field have opposite effect when compared with temperature.

**Fig 12:**
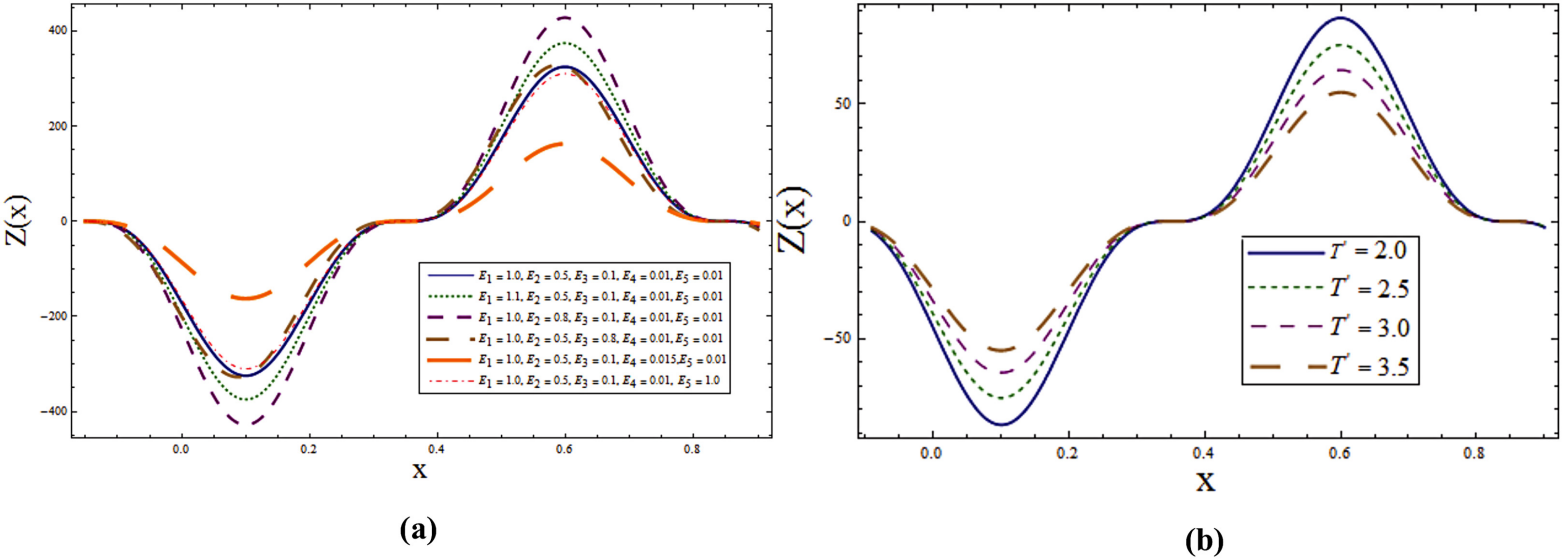
**(a)** Effect of wall properties on ***Z*** when *Sr = Du = Sc = R = λ*_*1*_ = *M = 0*.*8*, *T*^*'*^ = *Pr = 1*.*0*, *K*_*1*_ = *0*.*1*, *Ec = 2*.*0*, *ε = 0*.*2* and *t = 0*.*1*. **(b)** Effect of ***T***^***'***^ on ***Z*** when *E*_*1*_ = *0*.*2*, *E*_*2*_ = *E*_*3*_ = *E*_*4*_ = *0*.*01*, *E*_*5*_ = *0*.*1*, *Sr = Du = Sc = R = λ*_*1*_ = *M = 0*.*8*, *K*_*1*_ = *0*.*1*, *Ec = 2*.*0*, Pr = 1.0, *ε = 0*.*2* and *t = 0*.*1*.

**Fig 13:**
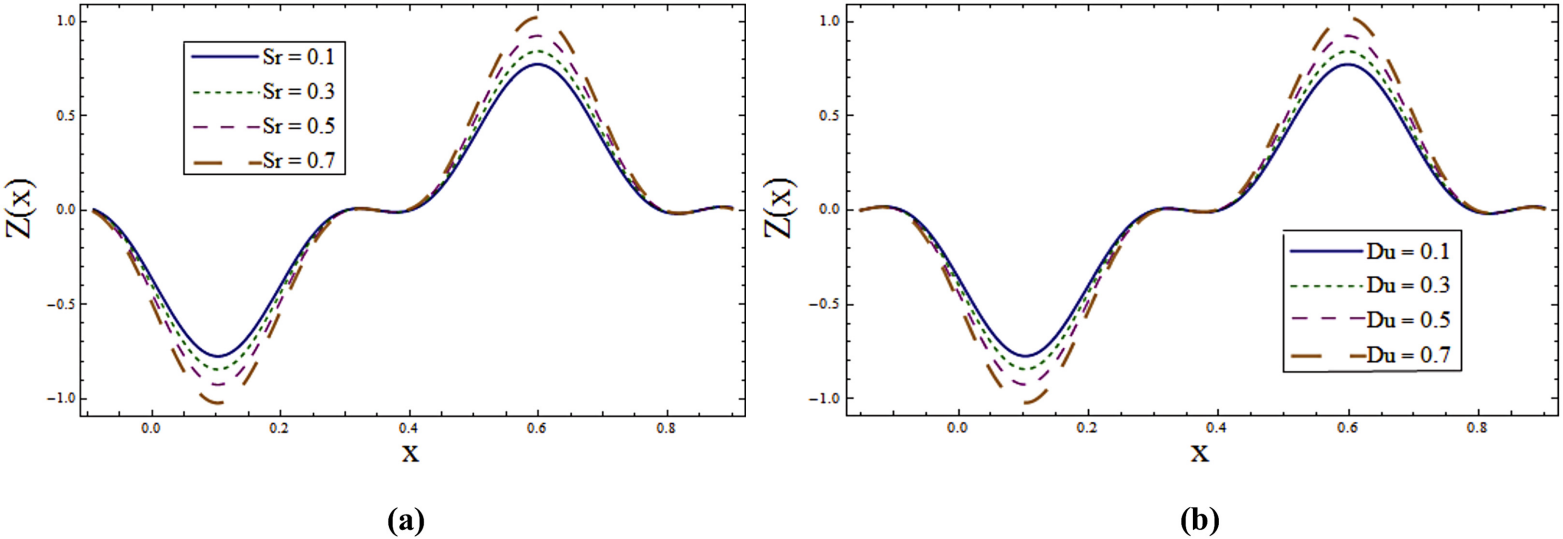
**(a)** Effect of ***Sr*** on ***Z*** when *E*_*1*_ = *0*.*2*, *E*_*2*_ = *E*_*3*_ = *E*_*4*_ = *0*.*01*, *E*_*5*_ = *0*.*1*, *Du = Sc = R = λ*_*1*_ = *M = 0*.*8*, *K*_*1*_ = *0*.*1*, *Ec = 2*.*0*, *T'* = Pr = 1.0, *ε = 0*.*2* and *t = 0*.*1*. **(b)** Effect of ***Du*** on ***Z*** when *E*_*1*_ = *0*.*2*, *E*_*2*_ = *E*_*3*_ = *E*_*4*_ = *0*.*01*, *E*_*5*_ = *0*.*1*, *Sr = Sc = R = λ*_*1*_ = *M = 0*.*8*, *K*_*1*_ = *0*.*1*, *Ec = 2*.*0*, *T'* = Pr = 1.0, *ε = 0*.*2* and *t = 0*.*1*.

**Fig 14:**
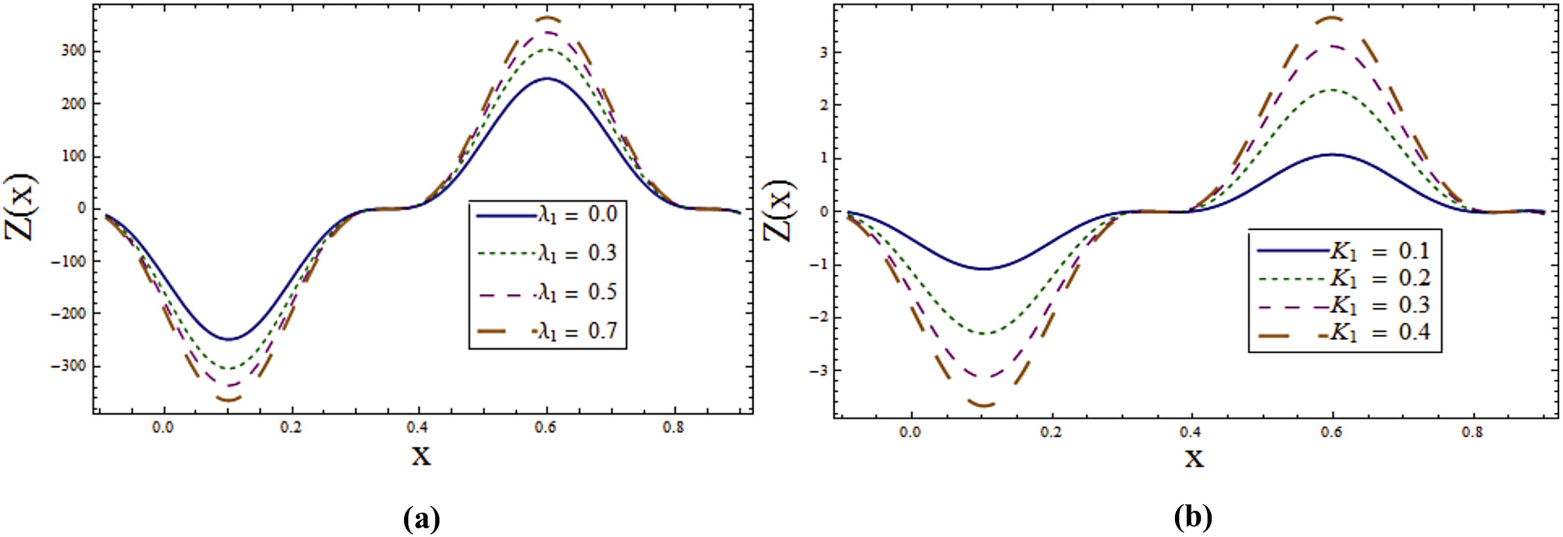
**(a)** Effect of ***λ***_***1***_ on **Z** when *E*_*1*_ = *0*.*2*, *E*_*2*_ = *E*_*3*_ = *E*_*4*_ = *0*.*01*, *E*_*5*_ = *0*.*1*, *Sr = Sc = R = Du = M = 0*.*8*, *K*_*1*_ = *0*.*1*, *Ec = 2*.*0*, *T'* = Pr = 1.0, *ε = 0*.*2* and *t = 0*.*1*. **(b)** Effect of ***K***_***1***_ on ***Z*** when *E*_*1*_ = *0*.*2*, *E*_*2*_ = *E*_*3*_ = *E*_*4*_ = *0*.*01*, *E*_*5*_ = *0*.*1*, *Sr = Sc = R = Du = λ*_*1*_ = *M = 0*.*8*, *Ec = 2*.*0*, *T'* = Pr = 1.0, *ε = 0*.*2* and *t = 0*.*1*.

**Fig 15:**
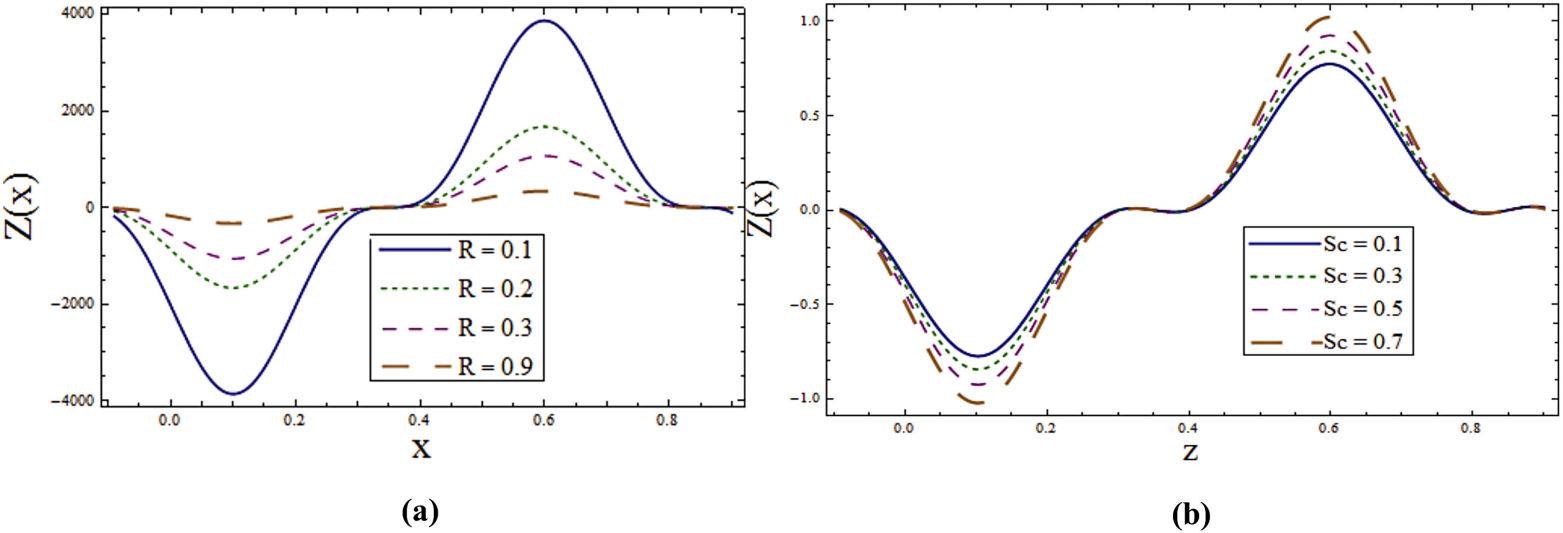
**(a)** Effect of ***R*** on ***Z*** when *E*_*1*_ = *0*.*2*, *E*_*2*_ = *E*_*3*_ = *E*_*4*_ = *0*.*01*, *E*_*5*_ = *0*.*1*, *Sr = Du = Sc = λ*_*1*_ = *M = 0*.*8*, *K*_*1*_ = *0*.*1*, *Ec = 2*.*0*, *T'* = Pr = 1.0, *ε = 0*.*2* and *t = 0*.*1*. **(b)** Effect of ***Sc*** on ***Z*** when *E*_*1*_ = *0*.*2*, *E*_*2*_ = *E*_*3*_ = *E*_*4*_ = *0*.*01*, *E*_*5*_ = *0*.*1*, *Sr = Du = R = λ*_*1*_ = *M = 0*.*8*, *K*_*1*_ = *0*.*1*, *Ec = 2*.*0*, *T'* = Pr = 1.0, *ε = 0*.*2* and *t = 0*.*1*.

Behavior of heat transfer coefficient *Z* for various parameters is shown in the Figs 12–15. The heat transfer coefficient is represented by *Z(x) = η*_*x*_*θ*_*z*_*(η)* which defines the rate of heat transfer or heat flux at the walls. As expected *Z* shows an oscillatory behavior which is because of the propagation of sinusoidal waves along the channel walls. Fig 12a and 12b explore the effect of wall properties and *T*^*'*^ on *ϕ*. It can be noticed that there is an increase in rate of heat transfer for *E*_*1*,_ *E*_*2*_ and *T*^*'*^ whereas decrease in the heat transfer rate is observed for *E*_*3*,_ *E*_*4*_ and *E*_*5*._ Effects of *Sr*, *Du*, λ_1_ and *K*_*1*_ on *Z* can be observed through Figs 13–14 respectively. By increasing these parameters the absolute value of *Z* increases. Effect of radiation parameter *R* can be seen in Fig 15a. Here increase in *R* enhances *Z*. It is clear from Fig 15b that rate of heat transfer is increasing function of Schmidt number *Sc*.

## Conclusions

Soret and Dufour effects in peristaltic motion of Jeffrey liquid in a channel with thermal radiation and porous medium are discussed in a rotating frame. The key findings of present study can be listed below.

1. Behaviors of wall parameters and Taylor number on axial and secondary velocities are opposite.
2. Behaviors of E_i_ (i = 3–5) on temperature are quite opposite to that of *E*_*1*_ and *E*_*2*_.
3. Temperature decreases for *T*^*’*^, *M* and *R*.
4. Similar behavior is observed for *Sr*, *Du* and *Sc* on temperature profile.
5. Concentration show opposite behavior when compared with temperature profile.
6. Heat transfer coefficient increases for *Sr*, *Du* and *K*_*1*_ but it decrease for *T*^*’*^.
